# Complete Genome Sequence of Escherichia coli BE104, an MC4100 Derivative Lacking the Methionine Reductive Pathway

**DOI:** 10.1128/MRA.00721-19

**Published:** 2019-07-11

**Authors:** Brian P. Anton, Richard D. Morgan, Benjamin Ezraty, Bruno Manta, Frédéric Barras, Mehmet Berkmen

**Affiliations:** a New England BioLabs, Ipswich, Massachusetts, USA; b Aix-Marseille Université, CNRS, Laboratoire de Chimie Bactérienne, Marseille, France; c SAMe Unit, Department of Microbiology, Institut Pasteur, Paris, France; Portland State University

## Abstract

In this announcement, we present the complete annotated genome sequence of an Escherichia coli MC4100 mutant strain, BE104. This strain has several methionine sulfoxide reductase gene deletions, making it ideal for studying enzymes that alter the redox state of methionine.

## ANNOUNCEMENT

Escherichia coli is the predominant prokaryotic model organism with two popular ancestral strains, K-12 and B (1). In general, E. coli K-12 is used for DNA manipulation and cloning, while E. coli B is used mostly for protein expression. There are many E. coli K-12 derivatives currently used in research and production laboratories worldwide, including MC4100, engineered by Malcolm Casadaban (2). Of the 801 completely sequenced E. coli genomes, only 33 are from K-12-derived laboratory strains, and of those, only 1 is derived from MC4100. MC4100 has been used extensively since its inception (3), resulting in thousands of publications. Its genome has been sequenced (4), and its relationship to other E. coli K-12 strains has been analyzed (5).

The strain BE104, a derivative of an MC4100 methionine auxotroph mutant lacking five methionine sulfoxide reductase (Msr)-encoding genes, was previously constructed in order to characterize an enzymatic system (MsrPQ) responsible for repairing proteins containing methionine sulfoxide in the bacterial periplasm (6). Briefly, BE104 was derived from MC4100, a methionine auxotroph mutant, by (i) a series of P1*vir* crosses to delete all cytoplasmic Msr-encoding genes (*msrA*, *msrB*, *msrC,* and *bisC*) by replacement with corresponding alleles from Keio knockout (KO) strains (7), (ii) selection for suppressor strains that could reduce methionine sulfoxide, and (iii) deletion of *msrP*, a consequently discovered periplasmic MsrP enzyme (5).

As BE104 is being used in our research and will be further engineered, we sequenced its genome using the Pacific Biosciences (PacBio) RS II sequencing platform, as described previously (8). BE104 was grown in standard rich medium (10 g/liter tryptone, 5 g/liter yeast extract, 5 g/liter NaCl, NaOH [pH 7.2]) at 30°C, and its genomic DNA (gDNA) was isolated using the Monarch gDNA kit (New England BioLabs). A SMRTbell library was constructed from 5 μg gDNA and sheared to ∼10 kb using a g-TUBE (Covaris). The library was sequenced on two single-molecule real-time (SMRT) cells using P6-C4 chemistry. The first cell yielded a 258-Mb sequence from 22,107 (15%) P1 reads, with a mean polymerase read length of 11,672 bases and a mean read insert length of 6,096 bases (180-minute data collection time). A second cell was sequenced to increase coverage, yielding 905 Mb of sequence from 63,262 (42%) P1 reads with a mean polymerase read length of 14,318 bases and mean read insert of 4,594 bases (240-minute data collection time). Sequencing reads were processed and assembled with the Pacific Biosciences SMRT Analysis v2.3.0 software using the HGAP3 protocol (expected genome size, 5 Mb; filters set to minimum subread length, 1,000 bp; minimum polymerase read length, 2,000 bp; minimum read quality, 0.80) and polished using Quiver (8). The 1.1 Gb of sequence assembled into a single closed circular genome of 4,775,122 bp with a mean coverage of 200-fold and a GC content of 50.8%. The assembled sequence was annotated using the NCBI Prokaryotic Genome Annotation Pipeline (PGAP) v4.8.

The following expected deletions were observed: *metB* (*O*-succinyl homoserine lyase) is disrupted by a 2-base deletion/frameshift; *msrA* is disrupted by insertion with a spectinomycin cassette; and the *msr* loci *msrB* (formerly *yeaA*), *msrC* (formerly *yebR*), and *msrP* (formerly *yedY*) are all deleted, as is *bisC* (biotin sulfoxide reductase) (9). As expected, the *yedV*::IS*2* insertion was not observed, as the *msrP*::*kan* deletion was transduced using the kanamycin marker from the Keio collection (JW1954), cotransducing the wild-type (wt) *yedV* allele.

This strain should be of general use to the research community for studying protein redox states and would be an important addition to the repertoire of sequenced E. coli genomes.

### Data availability.

The complete E. coli BE104 genome sequence has been deposited in GenBank with the accession number CP040643. The raw data are available in the NCBI Sequence Read Archive (SRA) with the accession number PRJNA544505.
